# The Fibrin Cleavage Product Bβ_15-42_ Channels Endothelial and Tubular Regeneration in the Post-acute Course During Murine Renal Ischemia Reperfusion Injury

**DOI:** 10.3389/fphar.2018.00369

**Published:** 2018-04-27

**Authors:** Dania Fischer, Christopher Seifen, Patrick Baer, Michaela Jung, Christina Mertens, Bertram Scheller, Kai Zacharowski, Rainer Hofmann, Thorsten J. Maier, Anja Urbschat

**Affiliations:** ^1^Department of Anesthesiology, Intensive Care Medicine and Pain Therapy, University Hospital Frankfurt, Frankfurt, Germany; ^2^Clinic of Internal Medicine III, Division of Nephrology, University Hospital Frankfurt, Frankfurt, Germany; ^3^Institute of Biochemistry I, Goethe University Frankfurt, Frankfurt, Germany; ^4^Clinic of Urology and Pediatric Urology, Philipps University of Marburg, Marburg, Germany; ^5^Department of Biomedicine, Aarhus University, Aarhus, Denmark

**Keywords:** FX06, renal ischemia reperfusion injury, endothelial activation, tubular regeneration, angiogenesis, primary mouse proximal tubular cells

## Abstract

Early and adequate restoration of endothelial and tubular renal function is a substantial step during regeneration after ischemia reperfusion (IR) injury, occurring, e.g., in kidney transplantation, renal surgery, and sepsis. While tubular epithelial cell injury has long been of central importance, recent perception includes the renal vascular endothelium. In this regard, the fibrin cleavage product fibrinopeptide Bβ_15-42_ mitigate IR injury by stabilizing interendothelial junctions through its affinity to VE-cadherin. Therefore, this study focused on the effect of Bβ_15-42_ on post-acute physiological renal regeneration. For this, adult male C57BL/6 mice were exposed to a 30 min bilateral renal ischemia and reperfusion for 24 h or 48 h. Animals were randomized in a non-operative control group, two operative groups each treated with i.v. administration of either saline or Bβ_15-42_ (2.4 mg/kg) immediately prior to reperfusion. Endothelial activation and inflammatory response was attenuated in renal tissue homogenates by single application of Bβ_15-42_. Meanwhile, Bβ_15-42_ did not affect acute kidney injury markers. Regarding the angiogenetic players VEGF-A, Angiopoietin-1, Angiopoietin-2, however, we observed significant higher expressions at mRNA and trend to higher protein level in Bβ_15-42_ treated mice, compared to saline treated mice after 48 h of IR, thus pointing toward an increased angiogenetic activity. Similar dynamics were observed for the intermediate filament vimentin, the cytoprotective protein klotho, stathmin and the proliferation cellular nuclear antigen, which were significantly up-regulated at the same points in time. These results suggest a beneficial effect of anatomical contiguously located endothelial cells on tubular regeneration through stabilization of endothelial integrity. Therefore, it seems that Bβ_15-42_ represents a novel pharmacological approach in the targeted therapy of acute renal failure in everyday clinical practice.

## Introduction

Ischemia reperfusion injury (IR) leads to the development of acute kidney injury (AKI) characterized by a massive tubular cell death, mainly affecting proximal tubules within the outer stripe of the outer medulla (Bonventre, 2010). At the same time, endothelial cells in the renal vasculature undergo an early swelling, leading to the narrowing of the lumen (Flores et al., 1972; Brodsky et al., 2002). Additionally, loosening of interendothelial connections and the subsequent formation of interendothelial gaps with leak formation leads to dysfunction of the blood–tissue barrier (Basile, 2007), possibly influencing recovery of the affected segments. These renal alterations rapidly evoke a process of regeneration, leading to restoration of both normal tubular and endothelial architecture. Hence, endothelial injury and capillary loss in the immediate vicinity of damaged renal epithelial tubules seem to affect tubular recovery and are therefore of particular interest.

In this context, vascular endothelial (VE)-cadherin is one of the most important endothelial anchor proteins which is connected to the actin-based cytoskeleton and one of the key molecules integrating and tightening endothelial cell junctions maintaining the vascular barrier integrity (Dejana et al., 2001; Broman et al., 2007; Vestweber, 2008). In turn, endothelial cell VE-cadherin interacts with the β_15-42_ sequence of fibrin (Bach et al., 1998; Gorlatov and Medved, 2002; Petzelbauer et al., 2005). Bβ_15-42_, also known as FX06, is a naturally occurring 28 amino acid cleavage product of fibrin. Its biological properties were first described in Petzelbauer et al. (2005). In supra-physiologic doses, this peptide has proven its efficacy to prevent VE-cadherin disruption, and thus micro-vascular dysfunction, finally achieving an organo-protection in myocardial IR injury in rodents (Petzelbauer et al., 2005), pigs (Roesner et al., 2007) as well as in humans (Atar et al., 2009), and recently in a clinical case of vascular leak syndrome during Ebola virus disease (Wolf et al., 2015).

Within murine ischemic kidneys, fibrinogen α, β, and γ chain mRNA were identified to be significantly up-regulated (Krishnamoorthy et al., 2011). Furthermore, application of fibrinogen-derived Bβ_15-42_ peptide (3.6 mg/kg at reperfusion) demonstrated a therapeutic potential in terms of vascular congestion, kidney dysfunction, and proximal tubular damage (Krishnamoorthy et al., 2011). In a different study, Bβ_15-42_ proved to alleviate the development of IR damage in mouse models of ischemic AKI as well as renal transplantation (3 mg/kg shortly before and 5 min after reperfusion, respectively) (Sorensen et al., 2011). Own recent findings identified Bβ_15-42_ to exert beneficial effects during the early phase of murine IR injury through reduction of endothelial activation, invasion of neutrophil granulocytes as well as tubular damage (2.4 mg/kg) (Urbschat et al., 2014, 2015).

The aforementioned studies support the concept that supra-physiological presence of Bβ_15-42_ serves as a protective agent in renal IR injury. In the present study, we thus focus on the initiation of physiologic regenerative processes regarding endothelial as well as tubular epithelial cell repair and their linkage in sequential points of time in a murine model of renal IR.

## Materials and Methods

### Animals

50–60 days old male C57BL/6 mice (Janvier) were kept in the central research facility of the University Hospital Frankfurt. They were housed with water and food *ad libitum* in rooms with a 12 h light cycle. All procedures involving animals were approved by the Animal Care and Use Committee of the state of Hesse in Germany (V54-19c20/15-F35/04). Surgery and animal care were performed in accordance with the “Guide for the care and use of laboratory animals” (National Institutes of Health, volume 25, no. 28, revised 1996), EU Directive 86/609 EEC and German Protection of Animals Act.

### Interventional Groups

Mice were randomized into five groups (*n* = 8 per group): one control group without intervention (CTRL), two interventional groups with 24 h (saline 24 h) or 48 h (saline 48 h) of IR and intravenous (i.v.) application of saline immediately prior to reperfusion as well as two treated interventional groups with 24 h (Bβ_15-42_ 24 h) or 48 h (Bβ_15-42_ 48 h) of IR and i.v. application of the fibrin fragment Bβ_15-42_ immediately prior to reperfusion. Before the procedure, mice were anesthetized with an intra peritoneal (i.p.) injection of ketamine (100 mg/kg body weight) and xylazine (5 mg/kg body weight). After bilateral dorsal flank incision, renal arteries were clamped for 30 min under microscopic control using non-traumatic microvascular clamps with a jaw pressure of 85 g (Micro-Serrefine 8 mm, Fine surgical instruments). Bβ_15-42_ or saline was administered i.v. immediately prior to the removal of the clamp. Hereafter, the recovery of blood flow was visually inspected. Subsequently, incisions were closed in layers and mice were allowed to recover. Animals were sacrificed after 24 or 48 h of IR, complete kidneys carefully removed, and divided longitudinally into halves. One half of which was placed in 4% paraformaldehyde overnight, the other was stored at -80°C together with blood samples, pending further processing.

### Reagents and Dosage

Bβ_15-42_ (amino acid sequence GHRPLDKKREEAPSLRPAPPPISGGGYR) was kindly provided by Prof. Petzelbauer, Medical University of Vienna. Mice received a single bolus of 2.4 mg/kg body weight Bβ_15-42_ in a total volume of 100 μl via i.v. application immediately prior to reperfusion. The selected dose was based on a previous study in rodent myocardial IR evaluating various dosing regimens (Zacharowski et al., 2007) and according to previous own studies (Urbschat et al., 2014, 2015). 0.9% saline was used as control which has no effect on prevention of renal injury induced by IR nor on renal function recovery (Li et al., 2016).

### Isolation and Culture of Murine Proximal Tubular Epithelial Cells

Murine primary proximal tubular cells (PTCs) were obtained from C57BL/6 mice, as described earlier (Baer et al., 1997). In brief, after kidney removal, the tissue was minced and digested with collagenase/dispase. The digested fragments were passed through a 106-μm mesh. The cell pellet was preincubated with mouse immunoglobulin G (mIgG, 5 mg/ml) to block unspecific binding. To enrich PTCs, we used a rat-anti-mouse antibody against aminopeptidase M (CD13, GTX62507, GeneTex). Finally, cells were incubated with a bead-conjugated anti-rat secondary antibody and isolated by immunomagnetic separation applying the Mini-MACS system (Miltenyi). Isolated cells were grown in DMEM/HAM’s F12 (1:1) with GlutaMAX (31331-028, Gibco), supplemented with 10% FCS and 1% Penicillin/Streptomycin at 37°C and 5% CO_2_ in a humidified atmosphere.

### *In Vitro* Experimental Anoxia

For anoxia assays PTCs were plated in 96-well plates and chamberslides in DMEM containing 10% FCS. Subconfluent cells were exposed to anoxia for 1 h by either adding a layer of mineral oil (M5310, Sigma-Aldrich) on top of medium (designated as medium+oil) or completely immersed in sterile filtered mineral oil (designated as pure oil). Oil immersion mimicks *in vivo* ischemic conditions by restricting cellular exposure to oxygen and nutrients as well as limiting metabolite washout (Meldrum et al., 2001). After 1 h, mineral oil and medium was removed, cells were washed with PBS, and incubated either with or without addition of 6 μM Bβ_15-42_ to the medium with the start of reoxygenation for 24 or 48 h, which is in line with the animal trial.

### RNA Isolation and Real-Time-PCR

Total RNA was isolated from homogenized kidney samples (*n* = 8 per group) using TRI Reagent (T9424, Sigma-Aldrich) according to the manufacturer’s protocol. cDNA was synthesized using an iScript cDNA Synthesis kit (1708891, Bio-Rad Laboratories). Gene expression profiles from all samples (*n* = 8 per group) were assessed in duplicates by quantitative real-time polymerase chain reaction using a StepOne Plus Realtime PCR device (Applied Biosystems). Primer sequences are listed in **Table 1**. Relative changes in mRNA expression were calculated by normalizing the values to their corresponding 18S mRNA expression using the 2^-dCt^ method (Livak and Schmittgen, 2001).

**Table 1:** Real-time PCR primers used for the quantification of mRNA expression levels.

Genes	Forward (5′–3′)	Reverse (5′–3′)
18S	GTAACCCGTTGAACCCCATT	CCATCCAATCGGTAGTAGCG
ICAM-1	GAGCGGCGTCGAGCCTAGG	TCTCGTCCAGCCGAGGACCAT
P-selectin	ACGGTACCATGTCCCCAAGCT	CCAGCGCTCGTGGAATCTCTC
VE-cadherin	CGCGGTGGCTCCACTAAGCC	CTGCGATGGACTCTGCGCCC
Lcn-2	CCAGGGCTGGCCAGTTCACTC	TGGGTCTCTGCGCATCCCAGT
KIM-1	GCTCAGGGTCTCCTTCACAG	ACCACCCCCTTTACTTCCAC
VEGF-A	AAGGAGAGCAGAAGTCCCAT	CTTCATCGTTACAGCAGCCT
Angiopoietin-1	GAACCGAGCCTACTCACAGT	ATCAAGCTGCTCTGTTTGC
Angiopoietin-2	ACTACGACGACTCAGTGCAA	GTTCAGCAAGCTGGTTCCAA
Tie-2	AGTGGACCAGAAGGATGCAA	ATTGGTACAGTGGCACCTGA
Vimentin	AGATCGATGTGGACGTTTCC	TCCGGTACTCGTTTGACTCC
Klotho	GACTTTGCTTGGGGAGTTGT	CGGATGGCAGAGAAATCAAC
Stathmin	CTTGCGAGAGAAGGACAAGC	CGGTCCTACATCGGCTTCTA
PCNA	AATGGGGTGAAGTTTTCTGC	CAGTGGAGTGGCTTTTGTGA

### Protein Isolation and Western Blot Analyses

Cryosections of whole renal tissue (*n* = 8 per group) were lysed in ice-cold lysis buffer with phosphatase-inhibitor and protease-inhibitor. After centrifugation, supernatants were removed and total protein was determined applying the bicinchoninic acid method. 10% SDS-gels were loaded with 50 μg protein. Two consecutive samples of all of five intervention groups were analyzed on four membranes with Spectra Multicolor Broad Range Protein Ladder and MagicMark (26623 and LC5602, Thermo Fisher Scientific) as a standard. Nitrocellulose membranes were incubated with the following antibodies: Angiopoietin-1 (AF923, R&D Systems, dilution 1:1000), Angiopoietin-2 (GTX100928, GeneTex, dilution 1:1000), klotho (MAB1819, R&D, dilution 1:500), phosphorylated Tie-2 (AF3909, R&D, 1:200), Tie-2 (GTX107838, GeneTex, dilution 1:500), vascular endothelial growth factor (VEGF-A) (ab68334, Abcam, dilution 1:1000), VE-cadherin (CD144, 138101, BioLegend, dilution 1:1000), vimentin (MA5-11883, Thermo Fisher Scientific, dilution 1:1000), and β-actin (A5441, Sigma-Aldrich, dilution 1:10000). Above proteins were investigated in all samples (*n* = 8 per group). Proteins were immunodetected by enhanced chemiluminescence (Santa Cruz Biotechnology). Digitalization and evaluation of the blots were performed with a Kodak Imager (Carestream) and protein densitometry was determined using ImageJ. For densitometry of Angiopoietin-2, phosphorylated Tie-2, Tie-2, and VE-cadherin both bands were included. Original western blot images are shown in **Supplementary Figures S2–S4**.

### Immunohistochemical and Immunofluorescence Staining

Immunohistochemistry for Lipocalin-2 (Lcn-2), pTie-2, Tie-2, and immunofluorescence staining for stathmin and proliferation cellular nuclear antigen (PCNA) was performed. For specific immunohistochemical staining, slides were incubated with the following antibodies: Lcn-2 (MAB1857, R&D Systems, dilution 1:500), pTie-2 (Y1102/Y1100, AF3909, R&D Systems, dilution 1:100), Tie-2 (C1C3, GTX107838, GeneTex, dilution 1:500), and a non-primary antibody control for each antibody. For development, slides were incubated with catalyzed signal amplification system (REF K1500, DAKO). Counterstaining was done with hematoxylin for 10 min. Analyses were performed in a blinded manner. 10 fields of randomly taken pictures from each slide (*n* = 8 per group) were analyzed. Quantification of Lcn-2 was done using a script, programmed in Matlab (The Mathworks). The software determined the values of each pixel in the RGB space, thus allowing to measure the relative area occupied by brown pixels in relationship to blue pixels. Calculation was performed as previously published (Urbschat et al., 2014, 2015). In order to detect proliferation, slides were incubated with antibodies against stathmin (ab52630, Abcam, dilution 1:500) and Alexa Fluor 488-labeled secondary antibodies (A11070, Invitrogen, dilution 1:2000) as well as antibodies against PCNA (Santa-Cruz, sc-56, dilution 1:1000) and Alexa Fluor 546-labeled secondary antibodies (A11018, Invitrogen, dilution 1:2000). Immunofluorescence staining was performed in *n* = 4 per group. For analysis of F-actin fibers in primary mouse PTCs, cells were seeded in 8-well chamber slides and fixed with ice cold acetone and methanol. In order to visualize cell nuclei and actin fibers, the cells were stained with Hoechst (33342, Invitrogen by Thermo Fisher, dilution 1:5000) and rhodamine-phalloidin (P1951, Sigma, dilution 1:12000), diluted in 3% BSA in PBS for 1 h. Cells were washed in PBS and mounted with Glycergel (C0563, Dako) containing antifade. Images were acquired using the same settings for all samples. Brightness and contrast were adjusted individually for each image. Immunofluorescence staining of PTCs was performed once. Image analysis was performed with Image J software.

### Measurement of Serum Creatinine

Colorimetric estimation of serum creatinine was performed in duplicate (*n* = 8 per group) using the alkaline picrate method (Jaffé’s Method, LT-SYS Creatinin Jaffé, LT-CR 0121, Eberhard Lehmann GmbH). Absorbance was measured at wavelength 492 nm.

### XTT

Viability of murine PTCs was determined by a photometric assay using 2,3-*Bis*-(2-Methoxy-4-Nitro-5-Sulfophenyl)-2H-Tetrazolium-5-Carboxanilide (XTT). In brief, subconfluent cells in 96-well plates were exposed to anoxia for 1 h with subsequent reoxygenation for 24 h or 48 h, respectively. Hereafter, XTT reagent was added to each well as described by the manufacturer (A8088, Applichem) and incubation was continued at 37°C. Absorbance was measured in a microplate reader (ELx808, Bio-TEC Instruments, Inc.) at 450 nm vs. 630 nm. Experiments were conducted in triplicate in three independent experimental settings and are represented as mean ± SEM. The value of viability is expressed as percentage of viability of untreated control cells set as 100%.

### Statistical Analyses

Statistical analyses were performed applying GraphPad Prism^®^ 5.02 software (GraphPad Software). The distribution of variables was tested for normality using the Kolmogorov–Smirnov test. Accordingly, statistical significance was calculated using one-way ANOVA followed by Tukey’s multiple comparison test or Kruskal–Wallis test followed by Dunn’s *post hoc* test, where applicable. Significance of correlations was determined by Spearman’s test including all investigated groups. *p*-Values ≤ 0.05 were assumed as statistically significant. In the figures, horizontal lines within the boxes represent the medians, boxes represent the interquartile range (25–75%). Whiskers above and below the box indicate the 90^th^ and 10^th^ percentiles. The individual points that are plotted beyond the whiskers represent outliers, which were included in the statistical analyses.

## Results

### Reduced Endothelial Activation Upon Treatment With Bβ_15-42_

**FIGURE 1:**
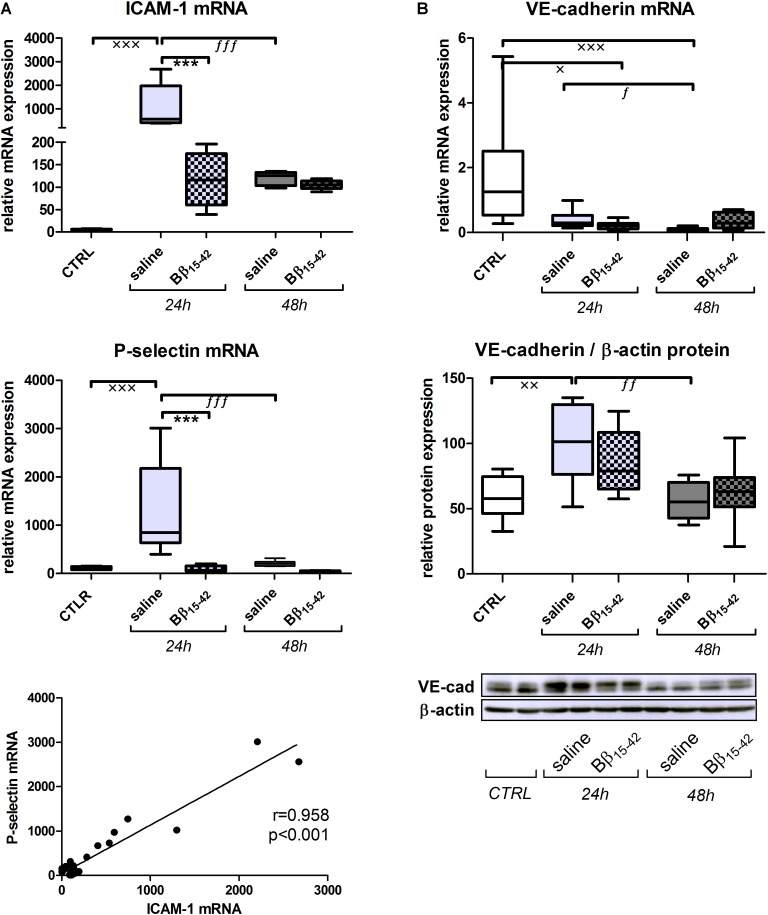
Bβ_15-42_ reduces inflammatory response and endothelial activation. To analyze the endothelial activation **(A)** and endothelial integrity **(B)** after IR injury, we performed RT-PCR analyses relative to 18S and western blot analyses relative to β-actin of relevant genes and proteins in kidney tissue homogenates (*n* = 8 per group for RT-PCR and western blot analyses). ^∗^ Significant difference between Bβ_15-42_ and saline treated mice at one point in time; x significant difference to CTRL; *f* significant difference in the time course within Bβ_15-42_ treated or saline treated mice. ^x/f^ *p* < 0.05; ^xx/ff^ *p* < 0.01; ^∗∗∗/xxx/fff^ *p* < 0.001.

First, we examined gene expressions of intracellular adhesion molecule-1 (ICAM-1) and P-selectin, as typical markers of endothelial activation (**Figure 1A**). ICAM-1 as well as P-selectin mRNA increased significantly at 24 h after IR in saline treated mice (*p* < 0.001) and to a significantly lower extend in Bβ_15-42_ treated mice (*p* < 0.001), although this differences abolished 48 h after IR (**Figure 1B**). Accordingly, we could observe a positive correlation between ICAM-1 and P-selectin (Pearson *r* = 0.958; *p* < 0.001) (**Figure 1A**). VE-cadherin represents a key molecule integrating and tightening endothelial cell junctions. Its mRNA decreased in both saline and Bβ_15-42_ treated mice after 24 and 48 h, compared to the control group. However, no marked difference was found between saline and Bβ_15-42_ treated mice at both points in time (**Figure 1B**). The corresponding VE-cadherin protein expression significantly increased after 24 h of IR but reached again levels of the control group after 48 h of IR, still no marked difference was found between saline and Bβ_15-42_ treated mice at both points in time (**Figure 1B**). In summary, we observed a significant endothelial activation in Bβ_15-42_ treated mice 24 h after IR.

### No Influence of Bβ_15-42_ on Kidney Injury Marker Within the Observed Points in Time

**FIGURE 2:**
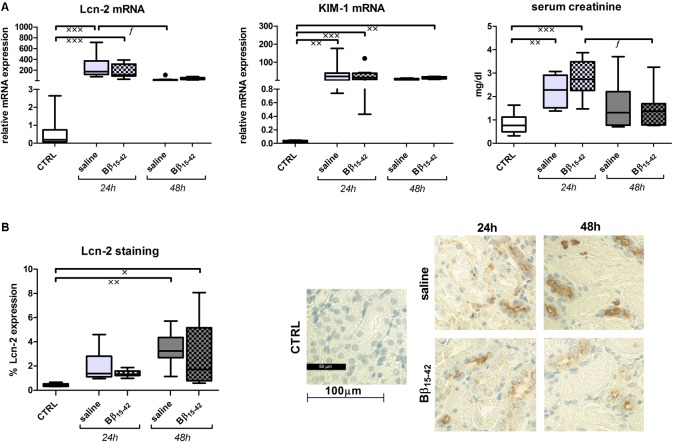
Evaluation of acute kidney injury (AKI). Tissue gene expression of the AKI markers Lcn-2, KIM-1, and serum creatinine **(A)**. Values are expressed in relation to the housekeeping gene 18S for mRNA analyses and in mg/dl for creatinine measurements. All experiments were performed in duplicate. Representative micrographs of Lcn-2 stained kidney sections of untreated control, saline and Bβ_15-42_ treated mice at 24 and 48 h IR **(B)**. Ten fields within the cortex and outer medulla in each group (*n* = 8) have been evaluated at 400-fold magnification. Quantification was performed using a script programmed in Matlab measuring proportional colored pixels in a defined relative area. Quantification was done by setting brown pixels into relationship to blue colored pixels. x significant difference to CTRL; *f* significant difference in the time course within Bβ_15-42_ treated or saline treated mice. ^x/f^ *p* < 0.05; ^xx^*p* < 0.01; ^xxx^*p* < 0.001.

Lipocalin 2 (Lcn-2), also called neutrophil gelatinase associated lipocalin (NGAL) and kidney injury molecule 1 (KIM-1) represent early biomarkers of ischemic AKI. Their mRNA expression was markedly elevated in both Bβ_15-42_ and saline treated mice at 24 and 48 h of IR, when compared to controls (*p* < 0.001) (**Figure 2A**). Likewise, levels of serum creatinine, which accumulates over time, rose significantly after 24 h in comparison to control mice but declined after 48 h of IR. However, none of the injury markers displayed differences between Bβ_15-42_ and saline treated mice at any point in time (**Figure 2A**). In order to quantify Lcn-2 tissue distribution, histological staining was performed and 10 randomly taken pictures from each slide were analyzed focusing on the cortex and outer medulla (*n* = 8 per group) (**Figure 2B**). Animals which received saline or Bβ_15-42_ exhibited increased Lcn-2 expression over time mainly in the distal tubule. Its expression reached a significant increase at 48 h of IR relative to the controls (saline: *p* < 0.01; Bβ_15-42_: *p* < 0.05) but no difference between Bβ_15-42_ and saline treated mice could be detected at any point in time (**Figure 2B**).

### Favored Angiogenesis Upon Treatment With Bβ_15-42_

**FIGURE 3:**
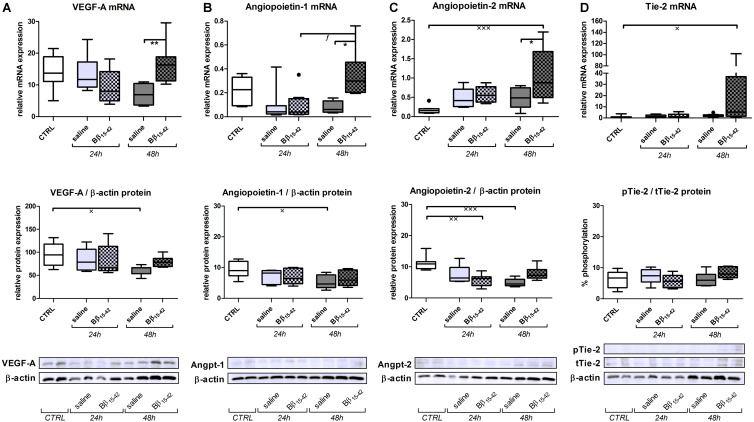
Bβ_15-42_ favors angiogenesis. In order to investigate angiogenetic signaling within IR injury, we performed RT-PCR analyses relative to 18S (upper graphs) and western blot analyses relative to β-actin (middle graphs and lower image) of relevant genes and proteins in kidney tissue homogenates (*n* = 8 per group) **(A–D)**. ^∗^ Significant difference between Bβ_15-42_ and saline treated mice at one point in time; x significant difference to CTRL; *f* significant difference in the time course within Bβ_15-42_ treated or saline treated mice. ^∗/x/f^ *p* < 0.05; ^∗∗/xx^*p* < 0.01; ^xxx^*p* < 0.001.

**FIGURE 4:**
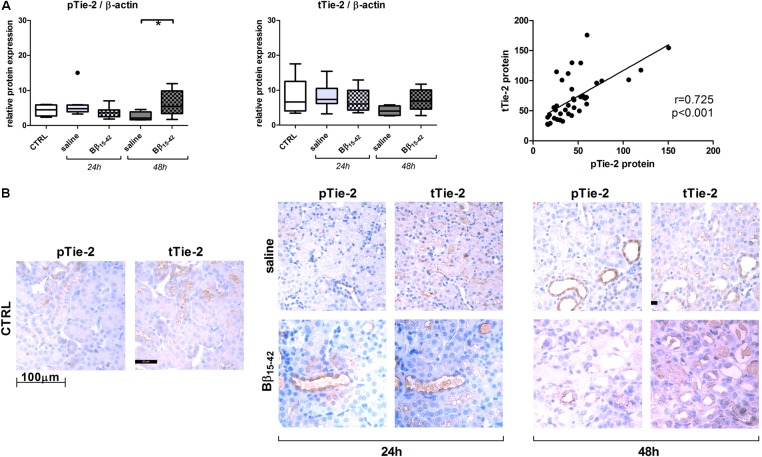
Protein expression and immunhistochemical staining of kidney sections on phosphorylated Tie-2 and total Tie-2. Protein expression of phosphorylated Tie-2 (pTie-2) and total Tie-2 (tTie-2) each relative to β-actin and their correlation **(A)**. Representative juxtaposed micrographs of phosphorylated Tie-2 (pTie-2) and total Tie-2 (tTie-2) stained kidney sections within the cortex and outer medulla of control, Bβ_15-42_ and saline treated mice at 24 and 48 h IR **(B)**. ^∗^*p* < 0.05.

Recovery from renal IR is associated with active angiogenesis, we thus investigated angiogenetic signaling by means of the following classical mediators of angiogenesis. Vascular endothelial cell growth factor (VEGF-A) mRNA and protein declined gradually over time upon IR. However, in Bβ_15-42_ treated mice, VEGF-A mRNA and protein was increased 48 h after IR compared to saline treated mice (**Figure 3A**). Similarly, Angiopoietin-1 mRNA displayed a higher expression in controls and decreased upon IR. Yet, Angiopoietin-1 mRNA and protein showed elevated expression at 48 h after IR in Bβ_15-42_ treated mice in comparison to saline treated mice (**Figure 3B**). In contrast, Angiopoietin-2 mRNA expression was lower in controls, and increased over time after IR. Again, after 48 h of IR, Angiopoietin-2 increased more firmly in Bβ_15-42_ mice compared to saline treated mice 48 h after IR. This is in contrast to the Angiopoietin-2 protein levels which decreased upon IR. Notably, Bβ_15-42_ counteracted the decrease in Angiopoietin-2 protein levels at 24 and 48 h of IR (**Figure 3C**). Tie-2 mRNA displayed similar levels in controls as well as saline and Bβ_15-42_ treated animals at 24 h after IR. However, a significant increase in Bβ_15-42_ treated mice after 48 h of IR versus control mice was seen. Western blot analyses of phosphorylated Tie-2 (pTie-2) put in ratio to total Tie-2 (tTie-2) did not display changes in the portion of phosphorylated protein (**Figure 3D**). When either phosphorylated Tie-2 or total Tie-2 protein expression was put in ratio to β-actin, we noticed a significant increase in pTie-2 protein expression in Bβ_15-42_ treated mice 48 h after IR (**Figure 4A**). Notably, immunohistochemical staining of pTie-2, and tTie-2 focusing on the renal cortex and outer medulla in control mice displayed a low expression of pTie-2 and tTie-2 mainly in the luminal surface of proximal tubules. Interestingly, pTie-2 and tTie-2 expression increased especially at 48 h of IR with accentuation rather in the collecting duct (**Figure 4B**).

### Increased Tubular Regeneration Upon Bβ_15-42_ Treatment

**FIGURE 5:**
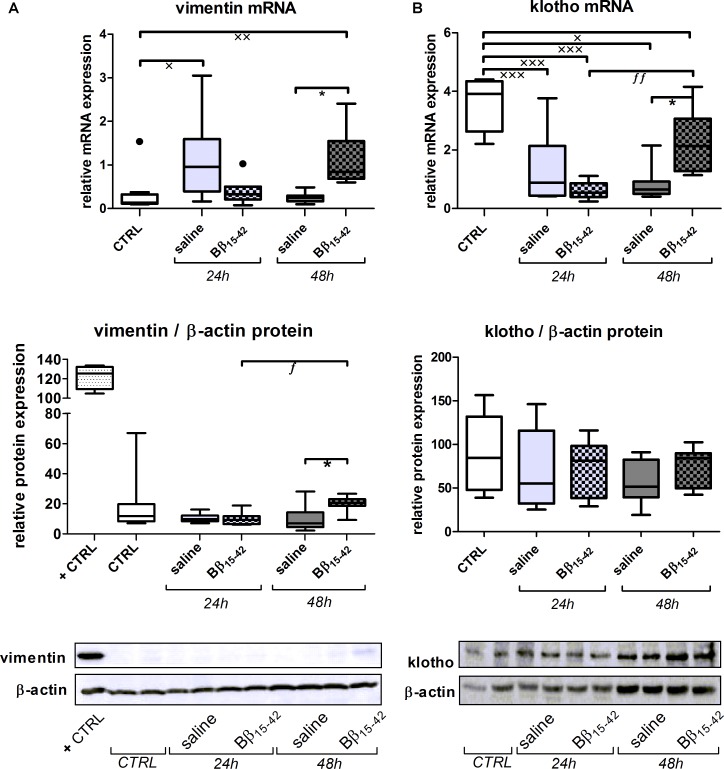
Increased tubular regeneration upon Bβ_15-42_ treatment. To provide an insight in tubular regeneration, proliferation and cell survival upon application of Bβ_15-42_ we performed RT-PCR analyses relative to 18S (upper graph) and Western blot analyses relative to β-actin (middle graph and lower image) of relevant genes and proteins in kidney tissue homogenates (*n* = 8 per group) **(A,B)**. Corresponding correlation (Spearman’s test) **(B)**. ^∗^Significant difference between Bβ_15-42_ and saline treated mice at one point in time; x significant difference to CTRL; *f* significant difference in the time course within Bβ_15-42_ treated or saline treated mice. ^∗/x/f^ *p* < 0.05; ^xx/ff^ *p* < 0.01; ^xxx^*p* < 0.001.

**FIGURE 6:**
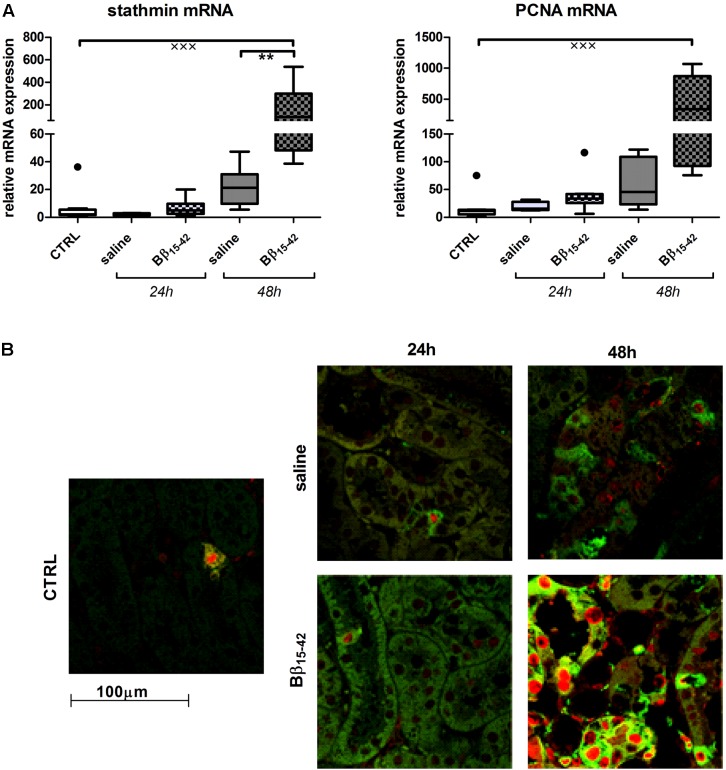
Increased tubular regeneration upon Bβ_15-42_ treatment. The effect of Bβ_15-42_ treatment on stathmin and proliferation cellular nuclear antigen (PCNA) was assessed by RT-PCR analyses relative to 18S in kidney tissue homogenates (*n* = 8 per group) **(A)** and immunofluorescence staining of stathmin (green) and PCNA (red) (400-fold magnification) in renal tissue (*n* = 4 per group) **(B)**. ^∗^Significant difference between Bβ_15-42_ and saline treated mice at one point in time; x significant difference to CTRL. ^∗∗^*p* < 0.01; ^xxx^*p* < 0.001.

Conclusively, we investigated biomarkers of tubular recovery after injury. Interestingly, vimentin exhibited markedly increased mRNA expression in Bβ_15-42_ treated mice after 48 h of IR compared to the saline control group (*p* < 0.05) (**Figure 5A**). Protein levels of vimentin were constantly very low and close to the detection limit throughout all samples with the exception of mice after 48 h of IR in Bβ_15-42_ treated mice (*p* < 0.05 versus saline). Since vimentin protein expression was rather week, we added a positive control which constituted of *in vitro* cultured mouse tubular epithelial cells known to co-express vimentin and cytokeratin as intermediate filament proteins (Baer et al., 1999) (**Figure 5A**). By contrast, the mRNA levels of the cytoprotective klotho decreased significantly in both Bβ_15-42_ and saline treated mice after 24 h of IR (*p* < 0.001). After 48 h of IR, klotho mRNA levels remained at a similar low level in saline treated mice but rose again significantly in Bβ_15-42_ treated mice (*p* < 0.05) (**Figure 5B**). Meanwhile, klotho protein expression remained relatively similar in CTRL and Bβ_15-42_ treated mice after 24 and 48 h of IR (**Figure 5B**). To further investigate regenerative and proliferative parameters, we performed gene expression analyses on stathmin and PCNA (**Figure 6A**). Both stathmin and PCNA mRNA exhibited markedly increased mRNA expression in Bβ_15-42_ treated mice after 48 h of IR compared to the saline control group (*p* < 0.001). Moreover, stathmin mRNA expression was significantly increased after 48 h of IR in Bβ_15-42_ compared to saline treated mice (*p* < 0.05), thereby indicating its pro-regenerative effect in kidney repair (**Figure 6A**). The effect of Bβ_15-42_ treatment on stathmin and PCNA was corroborated by immunofluorescence staining of stathmin (green) and PCNA (red) expression in renal tissue, focusing on the renal cortex and outer medulla (**Figure 6B**). Consistent with gene expression analyses, stathmin and PCNA expression showed that Bβ_15-42_ treated mice exhibited increased expression of stathmin and PCNA compared to the saline treated group after 48 h. Representative pictures out of four independent experiments are shown in **Figure 6B**.

### No Immediate Beneficial Effect of Bβ_15-42_ Treatment on PTCs

In order to examine a potential immediate effect of Bβ_15-42_ on PTCs we mimicked *in vivo* conditions in *in vitro* anoxia experiments using primary PTCs from the same mouse strain (C57BL/6). In these experiments, anoxia significantly decreased cell viability (measured by XTT) in pure oil treated cells compared to the control groups (24 h reoxygenation *p* < 0.001, 48 h reoxygenation *p* < 0.05) and moderately decreased cell viability in cells immersed in medium and oil layer atop (**Supplementary Figure S1**). For morphological analyses we performed Hoechst and phalloidin staining. In line with XTT results, we observed rarefaction of PTCs, implying preceding cell death. Furthermore, stress fiber formation decreased in mineral oil treated PTCs and was hardly detectable in pure oil treated PTCs. In contrast, actin aggregation tended to change from focal to more ubiquitous distribution upon increasing damage. However, no immediate benefit of Bβ_15-42_ on PTCs was noted (**Supplementary Figure S1**).

## Discussion

Vascular injury, in particular endothelial cell injury, participates in extent and maintenance of AKI. Over-expression of ICAM-1 and P-selectin by vascular endothelium of the ischemic kidney has been demonstrated to play a major patho-physiological role in the development of renal dysfunction (Kelly et al., 1994; Singbartl et al., 2001). IR injury also induces the generation of inflammatory mediators like cytokines and chemokines by both tubular and endothelial cells (Supavekin et al., 2003; Bonventre and Zuk, 2004). Accordingly, we observed a significant minor increase of ICAM-1 and P-selectin in Bβ_15-42_ treated mice, even though no effect on classical kidney injury markers could be sighted. Yet, these findings indicate that the applied treatment with Bβ_15-42_ effectively attenuated the extend of endothelial activation in our mouse model, which is in accordance with previous findings (Sorensen et al., 2011).

Given that fibrinogen β chain transcript levels were described to be one of the highest up regulated after kidney injury (Krishnamoorthy et al., 2011), modulation of endothelial integrity by means of Bβ_15-42_ application represents an attractive therapeutic approach. This holds true not only for early IR injury (Urbschat et al., 2014), but also for the post-acute period, which is characterized by the facilitation of endothelial and tubular repair mechanisms. In this context activation of angiogenesis plays a major role. Surprisingly, two independent studies demonstrated that neither mRNA nor protein expression of VEGF-A, the classic angiogenic factor, were increased in the post-ischemic rodent kidney (Kanellis et al., 2000; Basile et al., 2008), even though one would expect that hypoxia occurring during IR may stimulate the expression of pro-angiogenic molecules (Neufeld et al., 1999). This is in line with our observation, that both VEGF-A mRNA and protein expression declined over time in IR. However, its expression increased markedly merely in Bβ_15-42_ treated mice after 48 h of IR.

Aside from VEGF, Angiopoietins represent another family of endothelial specific growth factors (Satchell and Mathieson, 2003). Angiopoietin-1 is a secreted growth factor which binds to and activates the Tie-2 receptor tyrosine kinase. The latter regulates amongst others angiogenesis, endothelial cell survival, proliferation, and reorganization of the actin cytoskeleton. Angiopoietin-2, a further ligand of the Tie-2 receptor (Davis et al., 1996; Maisonpierre et al., 1997) was recently identified as partial agonist and antagonist for Tie-2 (Kim et al., 2000; Yuan et al., 2009). In our study not only VEGF-A but also Angiopoietin-1 and Angiopoietin-2 mRNA was significantly elevated in Bβ_15-42_ treated mice after 48 h with only a tendency for the corresponding protein expression, which might be due to temporally delayed protein expression. Noticeable discrepancy in Angiopoietin-2 mRNA and protein expression might be due to not immediate protein translation considering its function as partial agonist and antagonist for Tie-2 (Kim et al., 2000; Yuan et al., 2009). Yet, this indicates an earlier onset of vascular repair mechanisms which is particularly relevant owing to the fact that Angiopoietin-2 is only expressed at sites of vascular remodeling (Maisonpierre et al., 1997) and that application of synthetic Tie-2 agonistic peptide (Rubig et al., 2016) as well as VEGF (Kang et al., 2001) protects renal vascular function in experimental AKI.

Indeed, renal peritubular capillaries, which encounter endothelial swelling, capillary disintegration, and microvascular rarefaction during IR injury (Sutton et al., 2002; Molitoris and Sutton, 2004; Basile, 2007; Kwon et al., 2008) are morphologically and functionally closely connected with adjacent renal tubules. Yet, in *in vitro* co-cultures, a cross talk between primary human renal tubular and endothelial cells (Tasnim and Zink, 2012), and moreover, regulation of tubular recovery processes after injury by surrounding peritubular capillaries cells (Miya et al., 2011) have been discovered.

Above findings underline our observations as not only VEGF-A and angiopoietins are increased in Bβ_15-42_ treated mice after 48 h of IR, but also the intermediate filament protein vimentin and the cytoprotective protein klotho. Vimentin is typically detected in glomeruli, vessels, and interstitial cells, but not in tubular epithelial cells in the mature kidney (Holthofer et al., 1984; Witzgall et al., 1994). However, it becomes detectable in regenerating, mitotically active PTCs after IR (Nouwen et al., 1994) most prominently in the S3 segment 2 days post-ischemia (Witzgall et al., 1994). In this regard, it is also considered as a marker of epithelial dedifferentiation (Bonventre, 2003). Given that the fibrinopeptid Bβ_15-42_ binds to VE-cadherin (Zacharowski et al., 2006) we previously detected a significant alleviated loss of VE-cadherin in Bβ_15-42_ treated mice in very early IR injury (1 and 3 h after IR) (Urbschat et al., 2014). In this study however, no alteration in VE-cadherin expression in Bβ_15-42_ treated mice was noticed. Taking into account the short half-time of Bβ_15-42_ with 12–17 min in dogs and humans (Roesner et al., 2007), stabilization of VE-cadherin after single-shot application of Bβ_15-42_ might not be viewable in those later phases of IR. Nevertheless, VE-cadherin is linked not only by β- and α-catenin to cortical actin but also by γ-catenin and desmoplakin to the intermediate filament protein vimentin (Valiron et al., 1996; Kowalczyk et al., 1998). This linkage might irrespectively of our measured VE-cadherin expression influence the expression of vimentin to a certain extent, as vimentin expression rose in Bβ_15-42_ treated mice after 48 h of IR.

Along the same line, klotho is considered as cyto-protective mediator that is predominantly expressed in the kidney (Kuro-o et al., 1997). Immunostaining revealed constitutive klotho expression in the distal, cortical, and medullary collecting duct cells (Mitobe et al., 2005), whereas klotho mRNA and protein is also present in PTCs, although to a lesser extend (Hu et al., 2010a). Expression of both klotho mRNA and protein is known to significantly decrease during IR injury in the rat kidney, but rises gradually to nearly the control level during recovery (Sugiura et al., 2005; Hu et al., 2010b), suggesting a therapeutic potential in managing the ischemic challenge, too (Sugiura et al., 2005; Shi et al., 2016). As we detected a significant retrieval of klotho mRNA in Bβ_15-42_ treated mice after 48 h of IR, approaching constitutive expression. Klotho protein expression, still did only display a tendency to increased expression in Bβ_15-42_ treated mice 48 h after IR, which is presumably due to delayed protein translation.

Alike, immunofluorescence staining revealed a significant number of actively proliferating cells (stathmin and PCNA positive) within the renal cortex and outer medulla particularly 48 h after administration of Bβ_15-42_ compared to saline treated mice, which goes along with the respective mRNA expression in tissue homogenates. Increased detection of PCNA in nuclei of epithelial cells of the cortical proximal tubule has previously been described after renal IR (Witzgall et al., 1994). The maximum levels of expression were seen 48 h after IR mainly in the S3 zone, whereas in controls the number of labeled nuclei was considerably lower. In a further study immunofluorescent labeling of stathmin and PCNA demonstrated that the expression of stathmin increased markedly after 24 h and even more at 48 h of reperfusion (Zahedi et al., 2004; Jung et al., 2012), which is equally in accordance to our findings. As Bβ_15-42_ treated mice displayed higher expression of both, stathmin and PCNA 48 h after IR, this might be indicative for enhanced cell proliferation and regeneration within Bβ_15-42_ treated injured kidneys.

Still, because tubules and capillaries are simultaneously injured during IR injury, it is difficult to distinguish the primary initiating factors that drive endothelial-tubular crosstalk in these contexts. As we analyzed kidney tissue homogenates in gene and protein analyses both endothelial and epithelial cells were considered together, which certainly limits the deduction of conclusions.

Finally, as PTCs are known to be mainly affected by IR injury, *in vitro* experiments using primary murine PTCs from the same mouse strain (C57BL/6) were performed to mimic *in vivo* conditions. In these experiments, anoxic injury followed by reoxygenation significantly decreased cell viability and morphologically led to rarefaction of PTCs and reduced stress fiber formation. However, no direct effect of Bβ_15-42_ on PTCs could be observed. This observation reinforces the assumption, that Bβ_15-42_ promotes its protective effect through preservation of the endothelial integrity.

In the present study, angiogenetic factors as well as markers of tubular regeneration increased 48 h after IR injury upon treatment with Bβ_15-42_ suggesting a linkage between renal endothelial and tubular epithelial cells during cellular repair, as previously described in *in vitro* co-culture-assays. This cross-talk between injured tubular and endothelial cells is of particular interest and it is conceivable that these may either favor or prevent tubular recovery. Notably, the fibrinopeptid Bβ_15-42_ binds to VE-Cadherin, which in turn is relevant in preserving the endothelial barrier function.

As a physiologically occurring fibrinopeptide, Bβ_15-42_ represents a promising anti-inflammatory therapeutic agent channeling regeneration of the endothelium as well as tubules from ischemic injury. However, given the short half-time of Bβ_15-42_, during translation into efficient treatment in humans, a repeated application should be considered. In this regard, further investigations with focus on effected endothelial and tubular regeneration are warranted.

## Author Contributions

KZ and AU conceived and financed the study. DF, CS, CM, BS, and AU performed the analyses. DF, CS, PB, MJ, CM, RH, TM, KZ, and AU analyzed and interpreted the data and wrote the manuscript.

## Conflict of Interest Statement

The authors declare that the research was conducted in the absence of any commercial or financial relationships that could be construed as a potential conflict of interest.

## Supplementary Material

FIGURE S1 No immediate effect of Bβ_15-42_ on primary murine proximal tubular cells. Exemplary image of mouse PTCs in passage 2 **(A)**. Viability was measured via XTT after 1 h of anoxia and 24 and 48 h of reoxygenation with or without addition of 6 μM Bβ_15-42_ to the medium at the time of reoxygenation (*n* = 3) **(B)**. Analysis of actin fibers in primary mouse PTCs at the same conditions (*n* = 1) **(C)**. ^x/f^ *p* < 0.05; ^xx^*p* < 0.01; ^xxx/f^ *p* < 0.001.

FIGURE S2 Original western blot images **Figure 1**.

FIGURE S3 Original western blot images **Figure 3**.

FIGURE S4 Original western blot images **Figure 5**.
