# Supplementary figures and images for: The Fibrin Cleavage Product Bβ15-42 Channels Endothelial and Tubular Regeneration in the Post-acute Course During Murine Renal Ischemia Reperfusion Injury
Source: Front Pharmacol. 2018 Apr 27;9:369. doi: 10.3389/fphar.2018.00369 (PMC5934548; doi:10.3389/fphar.2018.00369)

Original western blot images figure 1

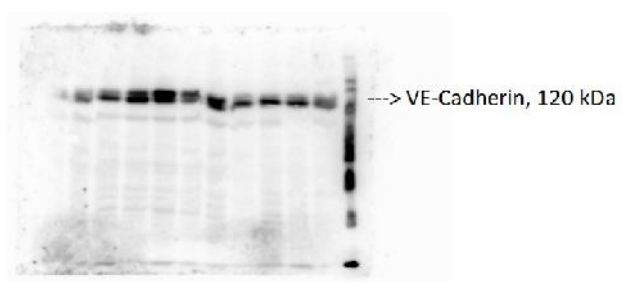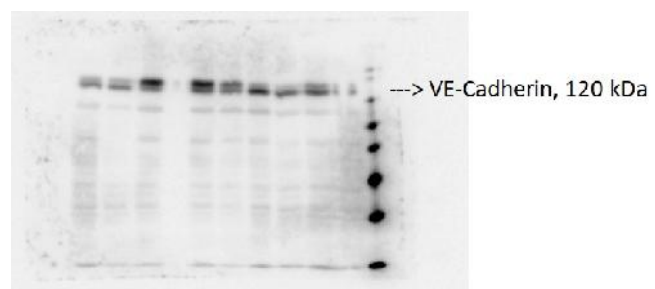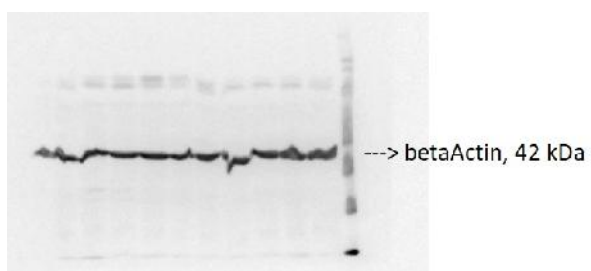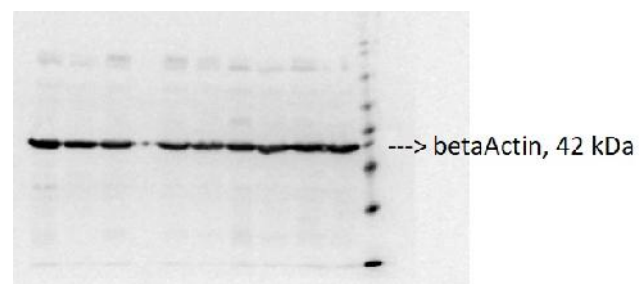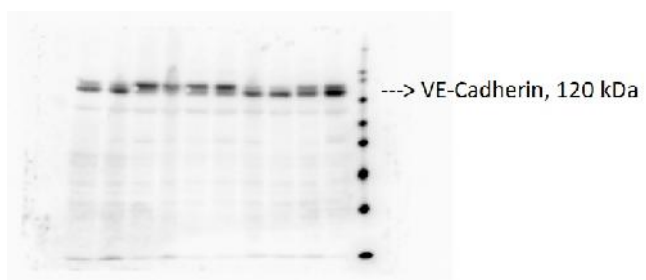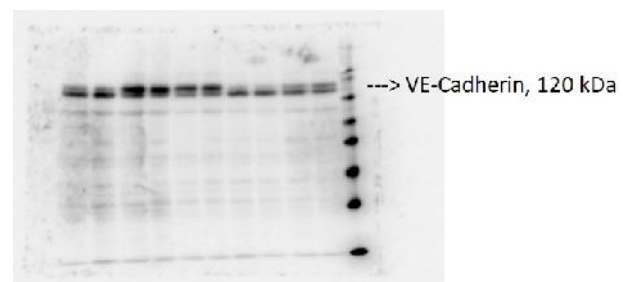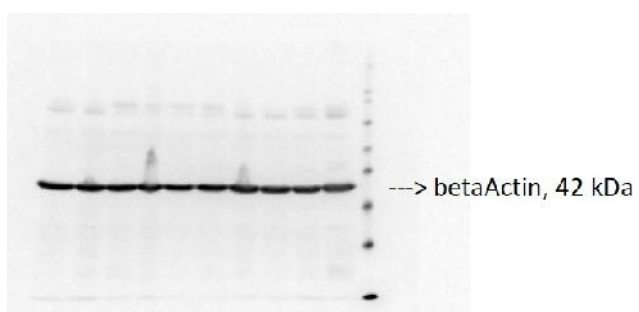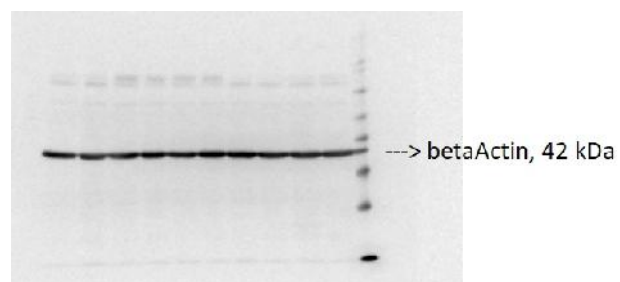

Supplement: FIGURE S2 — Original western blot images Figure 1. [file Image_2.PDF]

Original western blot images figure 5

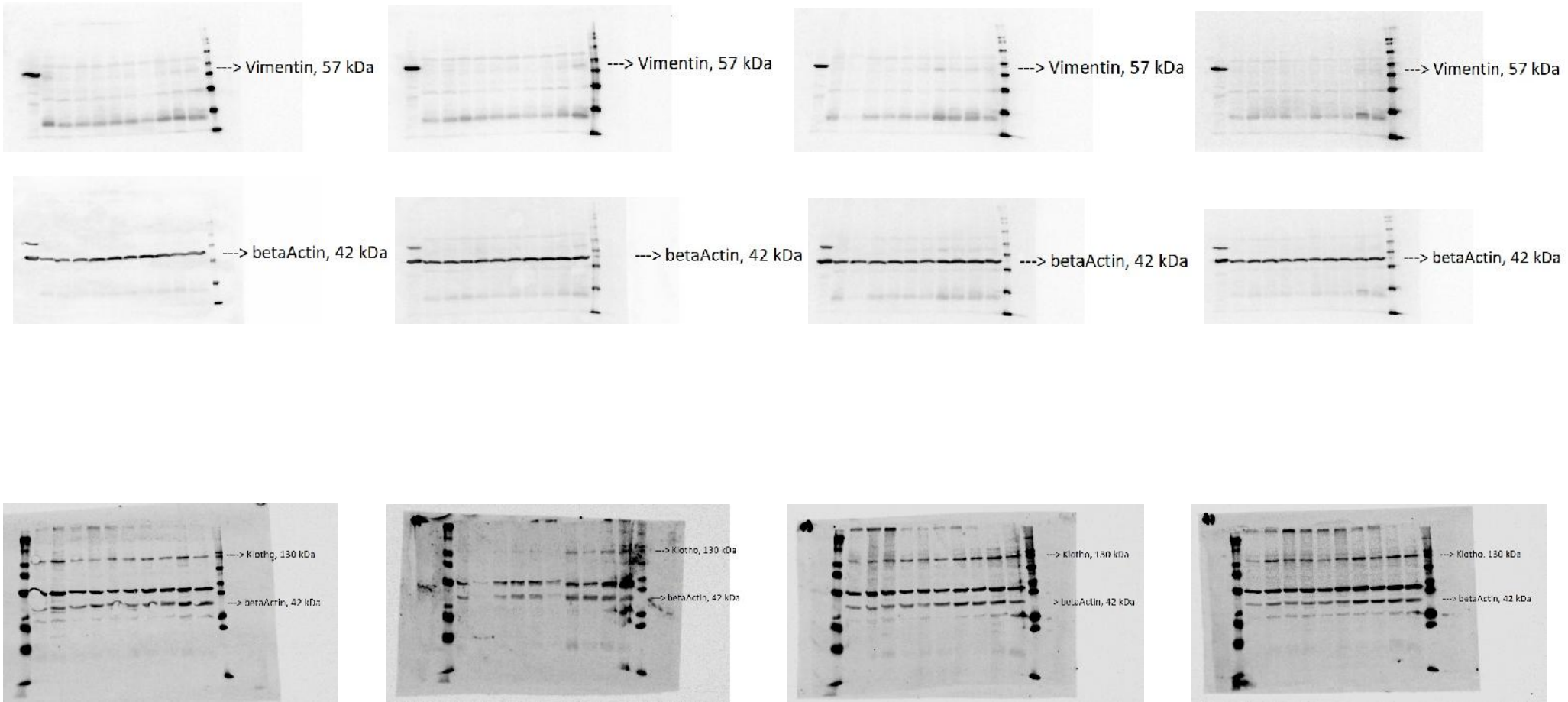

Supplement: FIGURE S4 — Original western blot images Figure 5. [file Image_4.PDF]
