# Supplementary material for: The Fibrin Cleavage Product Bβ15-42 Channels Endothelial and Tubular Regeneration in the Post-acute Course During Murine Renal Ischemia Reperfusion Injury
Source: Front Pharmacol. 2018 Apr 27;9:369. doi: 10.3389/fphar.2018.00369 (PMC5934548; doi:10.3389/fphar.2018.00369)
Supplement: FIGURE S3 — Original western blot images Figure 3. [file Image_3.PDF]

Original western blot images figure 3

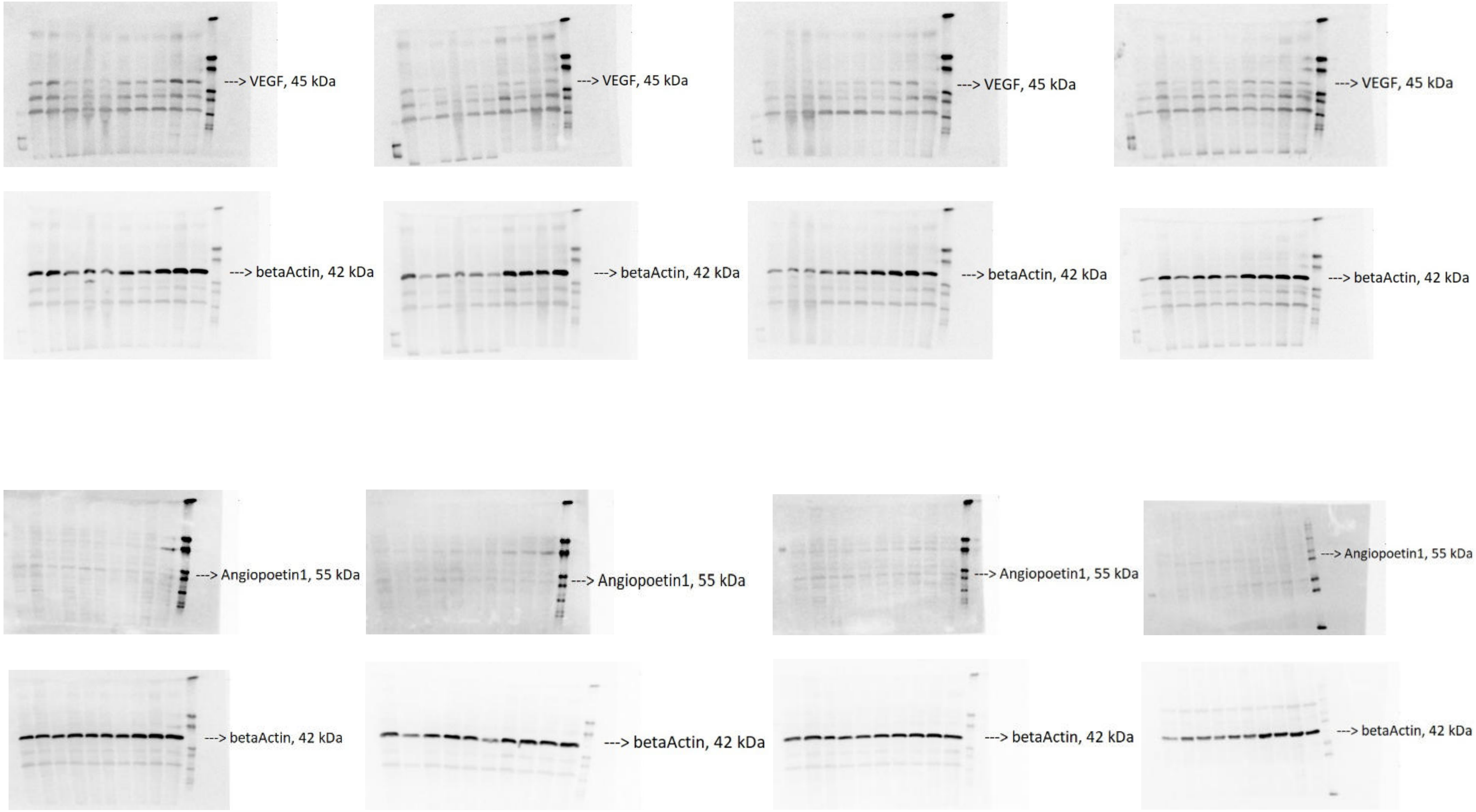

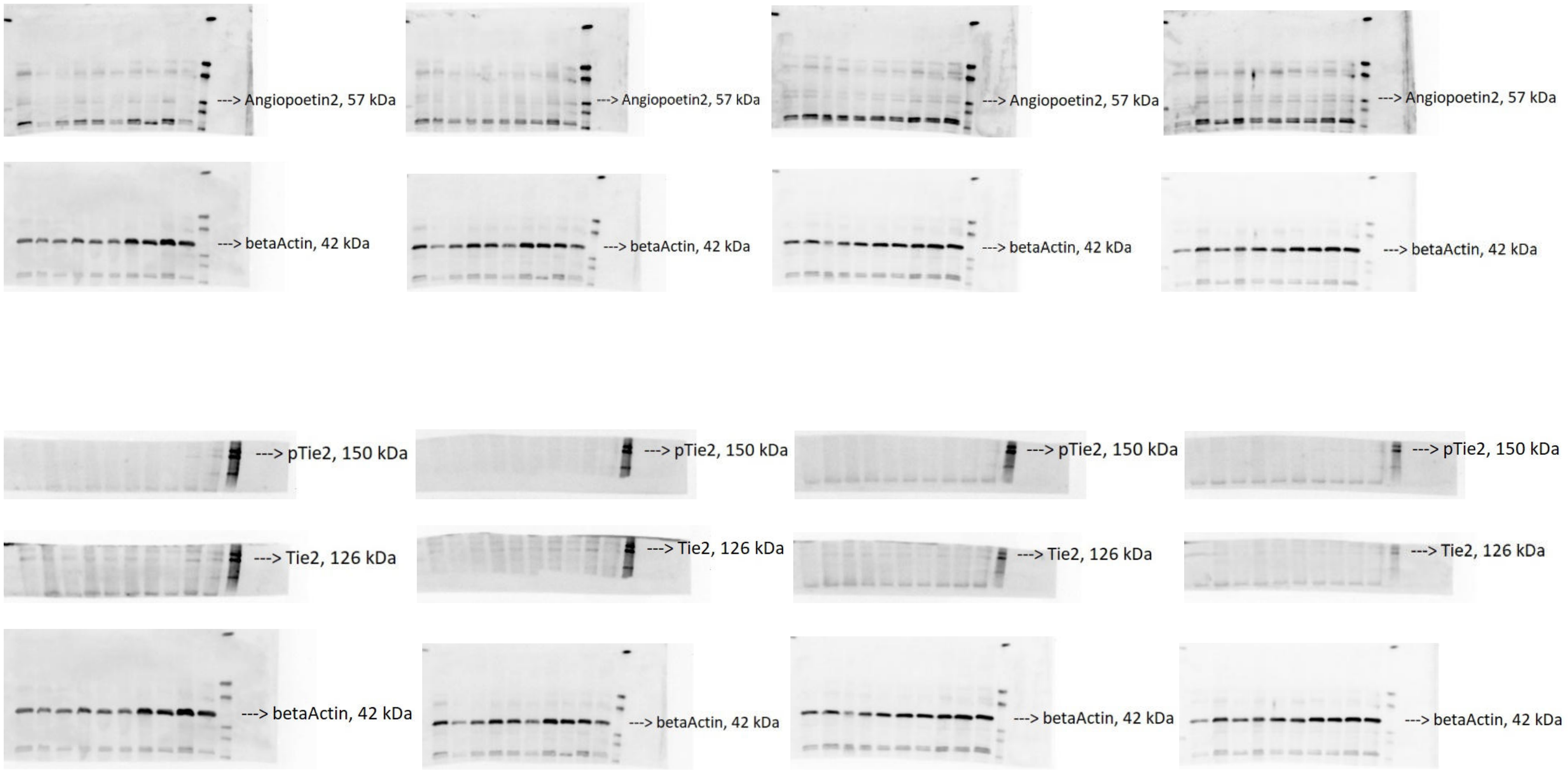

---> Angiopoetin2, 57 kDa
---> Angiopoetin2, 57 kDa
---> Angiopoetin2, 57 kDa
---> Angiopoetin2, 57 kDa
---> betaActin, 42 kDa
---> betaActin, 42 kDa
---> betaActin, 42 kDa
---> betaActin, 42 kDa
---> pTie2, 150 kDa
---> pTie2, 150 kDa
---> pTie2, 150 kDa
---> pTie2, 150 kDa
---> Tie2, 126 kDa
---> Tie2, 126 kDa
---> Tie2, 126 kDa
---> Tie2, 126 kDa
---> betaActin, 42 kDa
---> betaActin, 42 kDa
---> betaActin, 42 kDa
---> betaActin, 42 kDa
